# Qualitative assessment of transcultural psychotherapy by adolescents and their migrant families: Subjective experience and perceived effectiveness

**DOI:** 10.1371/journal.pone.0237113

**Published:** 2020-08-06

**Authors:** Emilie Carretier, Léa Grau, Malika Mansouri, Marie Rose Moro, Jonathan Lachal

**Affiliations:** 1 Maison de Solenn, Cochin Hospital, Paris, France; 2 Inserm, Centre de Recherche en Epidémiologie et Santé des Populations, Team DevPsy, Paris-Saclay University, Villejuif, France; 3 Unité Transversale de Recherche Psychogenèse et Psychopathologie, Paris 13 University, Villetaneuse, France; 4 Safe Childhood Association, Sauvegarde, Bobigny, France; International Telematic University Uninettuno, ITALY

## Abstract

**Objective:**

Adolescent migrants present psychological disorders more frequently than the corresponding host population but their access to care and to follow-up are less effective. The French method of transcultural psychotherapy (TPT) was conceived to respond to these problems. Our objective is to assess how these adolescents and their families perceive the experience and effectiveness of TPT.

**Method:**

We conducted semistructured interviews with the families of adolescents seen for TPT. The data were analyzed by a qualitative thematic methodology.

**Results:**

We spoke to 21 participants in 8 families. The families came to TPT with a sense that the teen's current treatment was at an impasse. During the follow-up, they noted that family communication and relationships had improved, as had their connection to their culture of origin. Besides commenting on what they perceived as limitations, families identified specific elements of TPT as therapeutic.

**Conclusion:**

The pronounced diversity of the group and the use of both multiperspective narration and an interpreter were specific elements driving the construction of a good therapeutic alliance, despite the initial barriers. Pursuit of the evaluation of TPT is essential to advance the psychiatric care of adolescent migrants.

## Introduction

Over the past century, the number of displaced persons, refugees, and migrants has grown exponentially [1]. According to the June 2019 statistical report of the United Nations High Commission for Refugees (UNHCR), more than 70 million people across the world had to flee their homes in 2018. The number of migrant adolescents, who either made the journey themselves (first-generation migrants) or were born in the host country to at least one parent born abroad (second-generation), continues to grow in Western Europe. Today, one adolescent in three born in France has at least one parent who comes from another country [2,3].

Adolescence is often defined as the period between the onset of puberty and the achievement of self-sufficienty [4]. The definition of adolescence as 10–24 years of age is widely used but continues to be shaped by culture and context [5,6]. This phase of life represents a relevant developmental stage during which important changes in social, behavioral, and emotional-motivational functioning occur [7,8]. For these characteristics, researchers indicate adolescence as a developmental phase at risk for the onset of mental disorders such as mood disorders, eating disorders or psychosis [9,10] but compliance with mental health treatments among adolescents is recognized to be poor [11]. Compliance with a treatment or therapy changes according to the quality of the alliance between the patient and the therapist. The concept of therapeutic alliance refers to the relationship of trust and collaboration that is established between a patient and his therapist [12].

Adolescent migrants could be a particularly vulnerable group because their displacement coincides with key developmental transitions. They may experience a combination of both the child to adult transition familiar from the culture of their home country, and the adolescent transition seen in host populations [13].

Studies show that there is a higher risk of internalizing problems, such as depression, anxiety and suicidal ideations, in migrant adolescent in Europe with 5 to 10% of the migrant adolescent affected. In most of the studies, Non-European migrants have a disadvantage in mental health compared with European migrant or native adolescents, whatever their origin or gender. [14–18].

Risk factors known to be especially hazardous for adolescents include out-of-home placement, loss or disappearance of a parent, exposure to traumatic events such as abuse, and damaging social environments [19]. Four or more of the risks just cited, dramatically increased the likelihood of psychiatric disorders [20]. Risks most commonly encountered by migrant adolescents include loss of a parent; separation from family members; exposure to multiple traumatic events, including war; and postimmigration stressors such as language difficulties, racial discrimination, and frequent moves [19,21]. The reasons for migration could be varied: economical (14%), family motives (32%), humanitarian (13%) [22]. However, whatever the type of migration, the traumatic potential exists with a risk of psychological disorder.

For comparable distress levels with the adolescents of natives of the host country, migrant adolescents are also less likely to seek help and are less often referred by professionals to mental health facilities. Finally, they face many barriers to mental health care, regardless of the host country and the organization of these services [16,23–25]. Studies have set forth the impact of difficulties in communication (understanding and expression) with healthcare providers, lack of knowledge of the healthcare system [26], and misunderstandings associated with the multiple cultural theories explaining the illness, which often conflict with Western medical theories [23]. These difficulties have a direct effect on the initiation, maintenance, and duration of care of migrant populations, and being a migrant is a risk factor for the premature termination of adolescent’s psychiatric treatment [27].

Given the many barriers that make care less accessible to a population at greater risk of mental disorders, improving our system of treatment is essential. Internationally, several models have sought to take cultural diversity into account in psychological treatment. Most of these initiatives have an indirect approach to care–focusing on training and supervision–, or offer cultural matching of patient and therapist [28–35]. Transcultural psychotherapy (TPT), as practiced in France, is an original therapeutic method designed to deal specifically with the difficulties encountered in psychiatric treatment of migrants. Development of this second-line therapy began in the 1980s, based on Georges Devereux's work in ethnopsychoanalysis [36–38]. It is based on an approach proposing two successive and complementary analyses—one anthropological, the other clinical—of the symptoms, rather than a simultaneous reading. Patients are seen most often with their families by a group of several therapists of diverse cultural origins and diverse fields of study, together with an interpreter-cultural mediator. The objective is to reestablish a dialogue that permits mutual understanding of the representations of the disease, its cultural etiology, and the ways of treating it [38–41].

The numbers of both qualitative and quantitative assessments of different types of psychotherapy have grown continuously in recent years [42–45]. Examination of client experiences is essential to advancing theoretical understanding of mediational processes in therapy, and qualitative research is an excellent way to investigate change processes in therapy [46–48]. The literature on transcultural psychotherapy exposes case studies [49], discuss the interests of the TPT [41,50] and give theoretical illumination [39,51] or practical explanations [52]. Nonetheless, as of now, no studies have directly assessed the care it provides. Our study will complement therefore the previous literature, being the first to assess the effectiveness of this therapy. It is an exploratory and qualitative first stage of ongoing research, aimed at assessing TPT qualitatively and quantitatively. In this article, we propose to explore how adolescent migrants and their families subjectively experience the teen's care in TPT. We aimed to look at the experience of care, its effectiveness, and the limitations perceived by the participants, as well as the aspects that they identified as therapeutic.

## Method

### Subjects and procedures

We recruited participants at a mental health center for adolescents in Paris (*Maison de Solenn*, Cochin University Hospital), which offers consultations with a group of transcultural psychotherapists. The participants were informed about this research either by a therapist at the TPT, at the end of a session, or by the referral nurse in a telephone call. A meeting was set up, during which adolescents and family members received clear oral and written information and provided written consent, in the presence of an interpreter. The researcher didn’t have relationship established prior to study commencement.

### Transcultural psychotherapy

The adolescents and their families are referred in second line by medical or social institutions after treatment as usual failed, when the referring team considers a transcultural clinical approach necessary to unlock a complex situation [40]. This care is usually organized in 90-minute sessions scheduled every 4 to 6 weeks (S1 File).

The patients are asked to come to the sessions with their families. They are seen by a group of several therapists of diverse cultural origins and an interpreter-cultural mediator with the same cultural background as the family. All conversations were translated word-for-word in both directions (the therapists' words to the patient and family, and the patient's and family's to the therapists). At least one professional from the referring institution, one who sees and knows the patient, is also asked to attend to provide continuity with their ongoing care.

The multicultural, multilingual group comprises around 10 therapists. A main therapist coordinates the sessions and calls upon the other therapists to speak. The latter formulated proposals, hypotheses, metaphors, or images, talking directly to the principal therapist, who rephrases these for the patient. Each therapist must be trained in decentering, that is, must be able to “not interpret the unknown in terms of the known” [53] and be able to accept that the center of the relationship is not only the therapist's but also the patient's center. They learn to distance themselves from their own point of view to be able to put themselves in the other's place and understand their perspective and their feelings [41,54].

The transcultural consultation is a flexible system, and its size and organization can be adapted to the situation. The presence of the interpreter is a key element in transcultural work, both at the linguistic level (understanding one another) and the symbolic level (recognizing the identity and singularity of the other). The principal therapeutic work is to open a dialogue on the different meanings of the disease, its cultural etiologies in the country of origin, and ways to perceive Western care. Subjects discussed in the consultation are the family's history, the history of their migration, and their encounter with European culture. The two targets of the therapy are the traumatic aspects of migration and the phenomenological splitting it may induce between the inside world (home) and outside (the host society) [38].

### Population and inclusion criteria

The population comprised adolescent migrants seen for TPT and their families. All were referred to TPT after one or more failed treatment attempts. Sampling was purposive. The adolescents included had to: a) be aged from 11 to 21 years, b) be first or second generation migrant, c) be accompanied at the psychotherapy sessions by family members (any of father, mother, or siblings), c) be able to express themselves in French or in their native language, and d) have been seen in TPT for at least 3 months. Adolescents with an acute decompensated psychiatric disorder were excluded.

We initially recruited ten adolescents and their family. Two were excluded due to unstable psychotic disorder.

### Data collection

The research was conducted through a semi-structured interview with the adolescent and his or her parents and siblings together, based on an interview guide developed by the research team (Table 1 and S1 Table). All interviews took place from November 2018 through February 2019 at the *Maison de Solenn* and were conducted by two residents in psychiatry (EC and LG). They were conducted in French or in the presence of an interpreter of the participants' principal language, if they did not all speak excellent French and if an interpreter participated to the therapy. The interview guide was flexible and adapted as appropriate during the course of the research. These lasted around an hour, were audio recorded with the participants' consent, and were subsequently transcribed verbatim in French. This research has been approved by the Research Ethics Boards of Sud Mediterranean IV.

**Table 1:** Semi-structured interview guide.

**Interview guide** • Can you tell me how did you arrived in transcultural psychotherapy consultation? • Can you tell me how your first session went and what impressions it left you with? • How was the continuation of care organized? Tell me the memories that stand out. • What seemed to you to be relevant and useful in the transcultural psychotherapy consultation, in terms of the initial problems? • What were the difficulties or negative points you encountered during the follow-up?

### Qualitative analysis

The interviews were analyzed by applying Interpretative Phenomenological Analysis (IPA), an iterative and inductive process [55]. The objective of IPA is to discover how subjects experience and give meaning to a phenomenon, by studying what they say about it [56]. The first stage began by reading each transcript in detail several times and coding it to identify the first themes. Meanings could appear at each reading. The recurrent themes and their connections were then identified in the different transcripts. These themes represent an understanding shared by the participants of the phenomenon being studied. This stage was more analytic since its objectives were to make sense of the associations between the themes and to show the similarities and differences between the accounts. The last stage involved producing a coherent and orderly presentation of the themes [55], that is, summarizing the set of experiences described and moving from a local theory of each interview to a general theory of the research question.

Results were analyzed as data collection continued. One author (EC), aided by NVivo 12 software, analyzed all of the interviews. Her analysis was triangulated by the other authors who are experts in qualitative method. The divergences were discussed in meetings with a team of experienced researchers. This triangulation made it possible to ensure that the themes identified accurately reflected the data. Discussing, clarifying, and, if necessary, modifying the themes contributed to improving the study's validity [55]. We reported our study in accordance to the Consolidated criteria for reporting qualitative research (COREQ) checklist (S2 File).

## Results

Data were collected in 8 interviews of 21 participants with 7 adolescents (mean age = 16,14), 10 parents and 4 siblings (Table 2). Half of the adolescents were first generation migrants. As no difference of experience was identified in the data, we did not differentiate the generation of migration in the presented results.

**Table 2:** Participants' characteristics.

Adolescent	Age	Sex	Main psychiatric symptom	Cultural problem	Participants (pseudonyms)	Country of origin	Generation of migration	Interpreter (therapy + interview)	Number of sessions	Therapy completed
P1	12	M	Depression	Cultural misunderstanding and family conflicts	Malick 3 sisters Mother	Guinea-Bissau	First	Yes	3	No
P2	21	F	Brief Psychotic Disorder	Cultural misunderstanding and family conflicts	Céline Mother	Sri Lanka	Second	Yes	8	No
P3	21	M	Depression	Traditional theory and family conflicts	Dylan Mother	Cameroon	First	No	18	Yes
P4	16	F	Brief Psychotic Disorder	Traditional theory	Ambre Mother	RD Congo	Second	Yes	3	No
P5	11	F	Depression	Traumatic migration and cultural misunderstanding	Nethmi Sister Mother	Sri Lanka	Second	Yes	13	Yes
P6	14	M	Selective mutism	Cultural misunderstanding	Natanaël Father Mother	Bulgaria	First	No	11	No
P7	18	F	Anorexia nervosa	Traditional theory and family conflicts	Aurélie Father Mother	China	Second	No	35	No
P8	20	F	Behavioral problems	Traditional theory and family conflicts	Jeanne Father	Benin/Martinique	First	No	16	Yes

Seven themes, classified under three superordinate themes, emerged from the analysis. *The personal experience and expectations of the families coming for transcultural therapy* dealt with the initial emotional aspects concerning this care and the initial expectations of the adolescents and their families. *The perceived effectiveness of the transcultural method* emerged from discussions of the changes perceived in intrafamily relationships and communication, their connections to both cultures, and the perceived limitations of the method. Finally, the participants identified *therapeutic elements* not directly related to the content of the consultations that played a role in the effectiveness of therapy: recourse to an interpreter, a group of therapists and multiperspective narration.

### 1. The personal experience and expectations of transcultural psychotherapy

This first superordinate theme describes the emotional feelings of the adolescents and their families during this treatment: the initial difficulties, emotions, doubts, and expectations related to the sessions.

**Theme 1.1. From daily ‴hell' to the implementation of this treatment: personal feelings of the adolescent and their families**.

The participants reported that before they came to this first appointment, they felt they were at an impasse, related to problems for which none of the different solutions proposed had been effective.

The families were surprised and impressed at their first meeting with the group of therapists. The group's size and shape differed substantially from the framework of the treatment they had previously experienced. Aurelie's father said: *"We were impressed by the system they had*. *That is*, *the form first*, *because we arrived in the room*, *we said*, *'wow*, *there's a lot of people here*!*' Then we were astonished that the people were so different*.*"* Participants needed time to become acquainted with the therapists before they could move past the first emotions of astonishment, even fear. The introductions of each of the therapists seemed reassuring to the families. Nethmi's mother noted that *"I asked myself*, *but … why are there so many people like that around me*? *But afterwards*, *when they each started to introduce themselves*, *that reassured me a little*.*"*

Some participants initially had doubts about the relevance of this care and the changes that might be possible. *"At the beginning*, *I was apprehensive*. *I said to myself that we were going to do [one of the things they recommended] and then that nothing was going to change*. *I didn't believe it*,*"* said one of Malick's sisters. On the contrary, the idea of being treated by an entire group was a relief for some, including Natanael's father: *"I said to myself*: *finally some specialists are going to listen to us*! *Well*, *anyway*, *a group of people who might be able to*… *to help us*.*"*

The emotional jolt described by the participants on discovering the group remained strong throughout the course of the psychotherapy. These transcultural sessions demanded an intense personal investment by each family member, sometimes inducing a backlash that had to be worked through later with the initial (first-line) therapist. Celine admitted, "*I must say that it's very emotionally charged*. *It takes me several days*, *even weeks*, *to recover from each session*.*"* According to Jeanne's father, *"a lot of energy was needed*. *A lot of passion too*. *… to be able to make it through these sessions and then a lot of hope to see my daughter and therefore my children get better*.*"*

**Theme 1.2. The personal expectations of the adolescents and their families**.

All of the interviewees reported their expectation that the therapy would help to resolve family conflicts, in particular, the relationships and communication between family members. Before the first session, they were expecting a space where it would be possible to reestablish the family dialogue that had become impossible in the private space of the home. Aurelie's mother wanted *"a place where we can talk*, *because at home*, *we can't*.*"* Expectations also included being able to talk about their culture of origin and their relation to it and to discuss cultural theories about the problems they faced. *"We talk about culture*. *We are looking for the impacts perhaps… some causes for the disease*,*"* according to Aurelie's father.

Participants often reported they came to these sessions with the idea of doing everything possible to help the child or the family. Sometimes, some even felt an initial obligation, an impression of constraint, of having no choice. Aurelie herself said: *"I don't have the impression I'm going for myself*. *I don't know*, *maybe for the family… In fact it's more of an obligation*.*"*

### 2. Families' perceptions of the effectiveness of transcultural psychotherapy

This second theme deals with the participants' perceptions of the effectiveness of TPT: the personal and interpersonal changes described by the families and the limitations of the therapy method that impeded change.

**Theme 2.1: Perceived personal and interpersonal changes during this psychotherapy**.

Early on, the family members participating in the sessions perceived a change, both in themselves and in the others. Some parents felt more confident in and acknowledged for their parental competence, including Nethmi's mother: *"They gave me self-confidence by telling me that you are capable of raising your daughters by yourself*. *They are the ones who gave me this confidence in myself*.*"*

Similarly, the adolescents felt more peaceful in their acceptance of their origins and the construction of their identity. They changed by integrating different points of view and enlarging their perceptions about the initial problem. Celine said, *"It helped me to accept what I cannot necessarily change*. *For example*, *my origins… it’s like that*, *it's part of me*.*"* She further noted that *"I'm learning other cultures at the same time*, *in fact*, *other ways of seeing things*.*"*

Beyond personal changes, interpersonal communication within the family also changed. TPT made it possible to reestablish a dialogue that until now had become impossible at home. Parents and adolescents succeeded in listening to each other, understanding each other, and getting to know each other again. *"What I liked*,*"* said Ambre's mother, *'it's that there are things my daughter never talked to me about that I discovered when I went to this consultation*. *She talks*.*"*

The participants reported calmer relationships, better understanding. Where family links had snapped, a form of continuity reappeared. According to Jeanne's father, *"We were able to explain to each other and that unlocked [something in her brother] about the relationship I have with her*.*"*

Finally, the participants described the modifications of the place that the culture of origin had in the adolescents' interactions and the construction of their identity. The latter can take place better through the *métissage* (hybridization) of their dual cultural affiliations, while the parents modify the way they transmit the culture of origin to adolescent born in France. Celine, for example, "*also ended up understanding that my parents' culture*, *it's a part of my identity*.*"* At the same time, her mother told us, *"that made her understand that she's not required to choose and that she can use both cultures*.*"*

**Theme 2.2: The perceived limitations of transcultural therapy**.

Although the participants had globally favorable opinions, they pointed out some limitations. Families felt that their initial lack of knowledge about this form of treatment, its objectives, and its methods had not been adequately considered. They wanted better information about the functioning and objectives of TPT. Jeanne's father suggested that *"from the start of the first session*, *[they ought to] perhaps explain the point of this method*.*"* Similarly, the interpreter reported that Celine's mother *"said that … at the first session she hadn't understood well*, *so it's necessary to explain why a little more*. *Because for example she came because she felt she was required to*.*"*

The group is described as flexible, and the people who compose it can change during the course of treatment. Nonetheless, the departures and arrivals of therapists in the group sometimes upset the families or raised questions among them. The value of including trainees in the consultation was sometimes misunderstood. According to Dylan's mother: *"So many faces*… *It would have been good to start with a team*, *it's not clear that they're there all the time*, *but at least*, *also finish with them*!*"* Celine mused, *"I wonder why [the trainees] are there*. *To listen*?*"*

The changes in the group and the long interval between sessions sometimes created the sense of a lack of continuity on several levels: between the sessions, in the maintenance of participation of family members from session to session, between the TPT and the external medical-social care. The patients sometimes perceived something "incomplete" about subjects mentioned from one session to another, as did Dylan's mother:

*And then often we might mention a subject and then not continue it at the next session*, *and then radically change directions at the meeting after*. *We didn't get to the bottom even the time after that*, *so several times there was a sense [that something] was incomplete*.

The discontinuity could also be due to the family. The absence of some members sometimes led to a sense of failure. According to Jeanne's father, *"on the family's side*, *it didn't work out at all because well*, *its … participation [by family members] petered out from the start*.*"*

The presence of the primary therapist at the session did not always diminish the feeling of compartmentalization of care. For some, including Dylan's mother, this psychotherapeutic work did not extend beyond the TPT sessions:

*I don't know what happened after the consultation but I had the feeling that after here*, *nothing else was done*. *Is there*, *for example*, *contact with a social worker who sees the child*, *is there contact with another doctor*? *If there was*, *I didn't really see it*.

### 3. The elements of transcultural psychotherapy identified as therapeutic

Here we describe the elements that the participants characterized as helpful in the therapeutic process

**Theme 3.1: Use of an interpreter**.

By using the parents' native language, the interpreters contributed to improving understanding between the adolescent and the family. Celine found it enlightening:

“*She translated with another meaning*. *And that was good, in fact, when you talk about translation*. *It's that she translated with a different meaning and so now I also understand that you can understand two different things with a person who speaks the same language*.”

This detour by the native language also seemed to bring the interpreter into the parent-adolescent relationship as a third party. Ambre noted: *"Because [there was] an interpreter who translated what I said*, *[we're not] directly in the process of communicating*, *both of us*, *emotionally*, *screaming*.*"*

With the interpreter present, some adolescents were relieved to be released from the role of family interpreter in which they are sometimes placed. This contributes to a readjustment of each person's role and place in the family. Dylan was one of these patients:

“*Arriving here in fact, what helped me it's that there was someone else who took over that role [of interpreter] … Finally each of us had our own role*. *Me I was me, my mother was my mother, there was an interpreter*. *And so, finally what worked, it's that we succeeded in putting each person's role where it actually belonged*. *Everyone was in their PLACE!*”

**Theme 3.2: The group, symbol of otherness**.

The group embodied many functions for the participants. A neutral third party in family conflicts, it participated the containment of emotions and support for speaking. According to Dylan's mother: *"There was anger in [my son]… It was important to be in a setting where he was able to expresses that anger*.*"* Aurelie's mother valued this for herself: *"There I can say all of my ideas*, *completely*, *and that lets me express myself*, *to say what I think*.*"*

The participants could directly discern the otherness of the group of transcultural psychotherapists, through the multiplicity of the therapists and of their occupations and cultures of origins. Their multicompetent and multicultural nature was rapidly identified as an advantage by the adolescents and the adults. As Aurelie's mother reported, *"We felt there were different cultures*. *It's like a team with different cultures and multiple skills*. *There's everything*, *there's psychiatrists*, *psychologists*, *nurses*.*"* The families felt reassured dealing with several therapists rather than only one. Each imagined more *"impartiality"* or *"neutrality"* than in a consultation with a single physician, because of the various viewpoints on the situation. As Jeanne's father said, *"At the session*, *what interested me … was the impartiality that might emerge due to the fact that there were 10 people; they couldn't all be bad or twisted*.*"* One of Malick's sisters liked the disagreement: *"Everyone is not necessarily in agreement with what the other said*, *in fact*, *so that's good too*.*"*

They pointed out the particular position of the principal therapist, which is defined as central, the guarantee of the organization and the smooth running of the session. Celine commented that: *"[What works in the consultation is] the teacher's questions*. *Because she interprets*, *but she also steers the session somewhat*. *There is a structure*.*"* Nonetheless, some participants were critical of the principal therapist's role in filtering. They regretted that the other therapists could not intervene more often and directly. Aurelie reported:

*I think that there are many [cotherapists] and so they can all ask different questions and as the questions come from different people*, *they should also be different questions*. *And there wouldn't be only the questions, the point of view*, *of the principal therapist that would be present*. *Even if they give their point of view as an image at the end, they don't interact very much with us*. *Even when they bring their images, they give them to the principal therapist*.

**Theme 3.3: The support for narrativity by multiperspective narration and sharing personal stories**.

The patients felt that the stories, images, and personal experiences recounted by the therapists helped them. Some of the therapists' accounts of their adventures, trials, and tribulations authorized patients to talk about their own journeys and draw parallels with their own histories. These different experiences described by the therapists enabled patients to feel less isolated and less alone in their distress. As Nethmi's mother told us, *"In fact [the principal therapist] told me my life history like a story*, *and that helped me a lot*!*"*

This form of psychotherapy allowed parents to recount the family history and enabled the adolescent to discover it, but also to talk about their culture of origin while being understood and not judged. The narrative was co-constructed as an exchange and mutual learning, the patients from the therapists but also the therapists from the patients. *"It's as if we were learning*. *It's as if they [the therapists] were learning from our culture …*. *We learn from them*, *a little from them*,*"* according to one of Malick's sisters. Being able to talk about culture also enabled parents to discover some cultural codes of France and then to "*se métisser*" alongside their children.

Nonetheless, the families did not receive the therapists' images and experiences passively. Active listening and important cognitive effort was required, as Natanael's father pointed out:

*For me, the last part, which is more or less the session of an image or a dream*, *I don't project myself into it at all*. *I'm not there at all, today with all the worries and constraints we have*, *I can't do it*. *They ask me to imagine, I can't anymore*.

## Discussion

Our study questioned 21 participants in 8 families undergoing TPT. These interviews enabled us to describe their personal experiences and their perceptions of the effectiveness of the therapy, as well as the elements they identified as therapeutic. On the basis of the accounts analyzed, we argue that a ***therapeutic alliance*** that was not initially evident is constructed progressively with the help of specific treatment elements clearly identified by our participants: the otherness of the group, the multiperspective narration, and the interpreter.

### The French transcultural psychotherapy method: The complex creation of an alliance

The very great majority of structured psychotherapies produce therapeutic benefits that are evaluated as equivalent today [57,58]. According to Despland, no definitive conclusions have been reached about the role of factors specific to each technique [58], but this is not the case for their common or shared factors [59,60]. Among the latter, the therapeutic alliance is simultaneously the most interesting and the most strongly related to the success of any type of therapy. It contributes to more than 50% of its total effectiveness [57,61,62]. There are three key components in the alliance: 1) the affective relationship between the patient and the therapist, 2) the consensus between the patient and the therapist on the global objectives of the treatment, and 3) the consensus between the patient and the therapist on the way to conduct the treatment and on the specific tasks related to the accomplishment of the treatment [63]. It is thus a transtheoretical factor identified as a predictor of the effectiveness of treatment, independently of the approach and the therapeutic technique [64].

Adolescent patients in general may show a poor alliance which may be characteristic of this specific phase of development [65]. Furthermore, the construction of a therapeutic alliance presents a difficult challenge and an important issue in clinical care for migrants [64], as shown by the high prevalence of early termination of care in this population [27]. Our results support this observation: families consult us in a context of ruptures and vulnerability (Theme 1.1), disappointment about previous treatments (Theme 1.1), and with numerous expectations of TPT (Theme 1.2). The meeting with the group of therapists is thus loaded with emotions: astonishment, fear, and a dose of distrust. Building a good therapeutic relationship in this context is not obvious.

These difficulties are enhanced by the families' **lack of understanding of the initial referral and of the objectives** of the TPT. An understanding between the therapist and patient about the general objective of the treatment is one of the three essential components of any therapeutic alliance [58,63,64]. Eckshtain et al. have thus proposed to transmit the basic principles of therapy to depressed adolescents to limit their early termination of care [66]. In TPT, this initial co-construction of a shared treatment project is all the more crucial because the involvement of individuals from different cultures [51,64,67] means that it must also deal with different representations of the disease and its care [28,68]. In the absence of clear information and the joint construction of treatment objectives, there is a risk that the working alliance will be poor, which in turn could compromise adherence to treatment [64]. For TPT, the establishment of a good alliance may therefore be initially at risk due to the families' lack of information about the indications and objectives of this treatment.

**Changes within the group**, departures, and absences, play a role in the families' poor identification with the therapists. These changes can sometimes weaken the affective links [58,63,64] built between the patients and the therapists in the group and thus imperil the overall therapeutic alliance. It is especially critical to consider this because migrant adolescent may well have faced separations, ruptures, and sociocultural discontinuity associated with migration and possible trauma; these can weaken the security of their attachments and their capacity to trust others [69].

Finally, some participants questioned the **structure of the treatment** and the role of the different therapists. In the TPT group, a principal therapist calls on others, including the cotherapists, to speak and rephrases the suggestions and comments they offer. Although the specific rules for assigning the floor, theorized in the past [38], can be reassuring for some patients, others experience them as an impediment to encountering the cotherapists. Some patients regretted that the principal therapist—perceived as a filter or a regulator—systematically controlled the interactions with the cotherapists. In this case, the affective bonds with some of them could be inadequate and could slow the establishment of the therapeutic alliance.

### The therapeutic elements identified in building the therapeutic alliance

Nonetheless, the clinical effectiveness of this method and the high attendance rate at sessions—estimated at more than 80% [70]—confirm the quality of the alliance during the course of treatment. The specificities of this method with a group of therapists, its *métissage*, and the use of interpreters and multiperspective narration are the drivers that enable the creation of the therapeutic alliance.

The families rapidly identify **the cultural and occupational diversity of the group** of therapists as an asset that facilitates the expression and **containment** of emotions. This visible otherness, an essential principle of the transcultural method used in Paris, was conceived to facilitate the establishment of a relationship between the therapists and patients [71]. According to Moro et al., the visible differences of the therapists "*allow patients to express their own singularity*, *both cultural and psychological"* [38]. Our results point in this direction. The families' experiences confirmed that the otherness of the group contributes to strengthening the therapeutic alliance.

**The use of the interpreter** also participates in "*staging otherness in this method"* [38] and therefore goes well beyond the translation of the discourse [72,73]. The interpreter translates each thing that is said, reinforces the therapeutic alliance, and serves as a key figure for adolescents in understanding their parents' representations [73–75]. The presence of a successful migrant helps in therapy supports the adolescent development which is even more complex through migration [75,76]. One of the adolescents talked about feeling freed from this role of translator for her parents for the first time and described it as an experience of relief. The use of an interpreter gave her access to different levels of understanding in her exchanges with her mother and let a third person into their relationship. Consistent with the literature, this teen described her strong identification with the interpreter, to the extent that this professional embodied the place that often falls to her in the family: that of the "go-between" from one universe to another [77]. The use of an interpreter can accordingly protect the teens from acculturative stress or internalization of depressive symptoms, as previously described for adolescents used as interpreters for their parents [78,79].

In the transcultural group, multiperspective narration is used especially to embody otherness and promote the emergence of a multiplicity of points of view on a single problem. The narration of the group's multiple perspectives authorizes patients to construct their own life stories and talk about their cultural representations, safe from criticism and certain of understanding [40]. The appropriate use of self-disclosure introduces different ways of thinking, makes the therapeutic relationship less dissymmetric, and thus strengthens the therapeutic alliance [80]. Face to face with otherness and confronted by numerous perspectives on the same problem, patients experience decentering.

### Limitations

This is the first qualitative study to assess TPT from the point of view of adolescents and their families in France. Nonetheless, it must be interpreted in light of its limitations. Our study is cross-sectional. Otherwise, the number of sessions of the participants was particulary varied. This difference between the participants can represent an element of discord in the results. A longitudinal study to assess changes over time in the experience and the therapeutic processes would be useful.

Moreover, we considered only the patients' and families' perspectives. The feelings and key events that the participants remembered and chose to describe may have been influenced by their desire to leave out some aspects of the therapy. Analyses crossing these data with the therapists' points of view could supplement and enrich our knowledge of the experience of TPT.

Finally, the limitation of recruitment to a single center specialized in transcultural therapy implies that the data are strongly influenced by this center's organization. The advantage was that we were able to assess the experience of the families receiving relatively homogeneous treatment. In the future, it would be useful to include families receiving care in different centers specialized in TPT to increase the generalizability of the results.

### Practical implications and perspectives for research

With access to participants' experiences, we are able to envision some improvements of this specific psychotherapeutic service.

It appears important to provide families with better information about the objectives of TPT from the first session, by involving them in the construction of clear treatment objectives that meet their initial needs and expectations.

Information about the organization of this treatment and especially the changes in the group from session to session—stability of a nucleus of therapists versus the mobility of the trainees—should be clearly established from the beginning. Absences in or departures from the group should be explained and justified.

Finally, some of our participants raised questions about the strongly structured nature of the group around the principal therapist, which is a factor of stability and *containment* in most situations. It remains to be considered whether the method should be adapted or if other types of treatment—individual, systemic therapy, etc.—should be considered in view of the questions raised about the model of the structured group.

TPT group managed to constitute a good relationship with patients despite the numerous families, organizational and cultural barriers raised in this study. In the context where mental care of migrant adolescents and families is a public health challenge, TPT is an emerging treatment that meets specific needs for this population of patients. Our study supports the proposition that TPT represents an additional treatment when usual care is ineffective. Qualitative and quantitative studies are needed to promote this psychotherapeutic care system.

## Supporting information

S1 File Description of the transcultural psychotherapy setting. (DOCX)

S2 File COREQ (COnsolidated criteria for REporting Qualitative research) checklist. (PDF)

S1 Table Semi-structured interview guide in French (original) and translated in English. (DOCX)
