# Supplementary material for: Qualitative assessment of transcultural psychotherapy by adolescents and their migrant families: Subjective experience and perceived effectiveness
Source: PLoS One. 2020 Aug 6;15(8):e0237113. doi: 10.1371/journal.pone.0237113 (PMC7410290; doi:10.1371/journal.pone.0237113)
Supplement: S1 File — (DOCX) [file pone.0237113.s001.docx]

**S1 File: Description of the transcultural psychotherapy setting**

**Patients encounter a group of therapists** of different cultural and linguistic backgrounds. The therapists may be trained in psychiatry, psychology, anthropology, sociology, history, linguistics or other scientific disciplines. The group is a variable-geometry setting. It size may vary from 2 to 10 or 15 therapists, depending on the patient origin and life history. For example, a specific smaller group is designed to receive unaccompanied minors who could be intimidated with too many therapists. On the contrary, a bigger group is designed for receiving most of Sub-Saharan African migrant families, as sickness is considered as a family and social concern and treated in the social group in most of African traditional societies.

One of the therapists is called the main therapist. He/she leads the encounter and distributes the floor. He/she may ask his/her co-therapists to provide comments, representations, symbolizations, metaphors, or interpretations of any aspects of the patients’ discourse. As all the words are directed to him/her, he/she has the power to reinforce or soften any proposal before giving it back to the patient. This rule is essential to protect the patients from aggressive, insensitive or chaotic interventions.

The group has four fundamental functions. At a general level, the group has a function of holding and psychic surrounding, which allows communication and elaboration for the parent often suffering from trauma, and for the child recognized in its otherness. At a cultural level, the group is the way of hearing and treating illness in traditional societies. It may be more comfortable for migrant persons to deliver their intimacy to a group than to a single therapist. Third, the multicultural group allows the process of decentring as it is a materialization of otherness. Lastly, the group proposes multiple different – and sometimes conflicting – ways of thinking illness. This process allows to access to the insight of patients, their psychic conflicts and complex representation of the world.

**The second main specificity** of the transcultural setting is the systematic use of interpreters speaking in the family’s mother-tongue. Depending on the family’s fluency, the interpreter may translate all the consultation word-to-word, or they may be solicited for some specific traumatic, emotional or intimate sentences. The presence of the interpréter is a key element in transcultural work, both at the linguistic level (understanding one other) and the symbolic level (recognizing the identity and singularity of the other). Research studies on working with interpreters in child mental health have shown that the interpreter has a great role in therapeutic alliance. They are a key figure for children as someone who can help to understand their parents’ representations, who make link between the inner world and the outside world. They are solicited as cultural informants and might be considered as co-therapists.

**The transcultural psychotherapy is a second line intervention**. Patients are referred to the consultation mostly by other physicians, psychologists, social worker or child protection services. Professionals refer patients when the experience difficulties in building up a trustful relationship and good communication, when they feel helpless when confronted with cultural specificities of illness representation, or when they doubt about the pertinence of their diagnosis and the patients’ compliance with treatment and services. Evaluation of the demands plays an important role. Referrals are discussed with the professionals, and sometimes their demand leads to an indirect consultation that should allow them to work in a more adequate way in their own consultation. When the group setting is proposed, follow-up should be provided by the initial care who is invited not only to participate in the evaluation, but also to take part in therapy sessions in the group setting.

**The main therapeutic work** lays on dialoguing between cultural meanings of the illness, traditional etiologies of suffering and western way of considering the medical care. The content of the consultations are narratives of the families’ history, of the migration, and of the confrontation to European way of living and understanding the world. The two targets of the therapy are traumatic aspects of the migration and psychic cleavage.

The group meets the patients with their families for one-hour sessions every seven weeks. The core family is invited with any member of the extended family or other people they feel close to, as well as the referring professionals.

**Originality** :
Culture has been a concern for a long time in psychiatry, and much research as well as many adaptations of therapeutic setting has been done over the world. Nowadays, many care settings designated for treating migrant families exist in America, Europe, or Australia. But none of them are laying on a multicultural group therapy. In Germany and Japan, where citizenship is based on an ethnic model, specific services devoted to each ethnic group are delivering care in the patient language. Australia and USA made the choice of addressing the language and developed the use of interpreters and cultural brokers who help the patient understanding the treatment. Canada, Italy, Belgium and UK developed specific settings that are indirect consultations and therapeutic mediations between professionals and migrant families. Finally, in many other countries, as for example in Northern Europe, transcultural psychiatry is underdeveloped. Most of the time only theoretical considerations and research studies exist.
