# Supplementary material for: Qualitative assessment of transcultural psychotherapy by adolescents and their migrant families: Subjective experience and perceived effectiveness
Source: PLoS One. 2020 Aug 6;15(8):e0237113. doi: 10.1371/journal.pone.0237113 (PMC7410290; doi:10.1371/journal.pone.0237113)
Supplement: S1 Table — (DOCX) [file pone.0237113.s003.docx]

**S1 Table: Semi-structured interview guide in French (original) and translated in English**

| **Guide d’entretien**   - Pouvez-vous me raconteur comment vous avez été adressé en consultation transculturelle? - Comment avez vous vécu votre première séance ? Quelles ont été vos impressions ? Racontez - Comment s’est organisée la suite de la prise en charge ? Racontez moi les souvenirs qui vous ont marqué. - Qu’est-ce qui vous a semblé utile et aidant dans la thérapie par rapport à vos difficultés initiales ? - Quelles ont été les aspects négatifs que vous avez ressentis ou identifies Durant votre suivi? |
| --- |

| **Interview guide**   - Can you tell me how did you arrived in transcultural psychotherapy consultation? - Can you tell me how your first session went and what impressions it left you with? - How was the continuation of care organized ? Tell me the memories that stand out. - What seemed to you to be relevant and useful in the transcultural consultation, in terms of the initial problems? - What were the difficulties or negative points you encountered during the follow-up? |
| --- |
